# Multimodal learning for fetal distress diagnosis using a multimodal medical information fusion framework

**DOI:** 10.3389/fphys.2022.1021400

**Published:** 2022-11-07

**Authors:** Yefei Zhang, Yanjun Deng, Zhixin Zhou, Xianfei Zhang, Pengfei Jiao, Zhidong Zhao

**Affiliations:** ^1^ College of Electronics and Information Engineering, Hangzhou Dianzi University, Hangzhou, China; ^2^ School of Cyberspace, Hangzhou Dianzi University, Hangzhou, China

**Keywords:** fetal heart rate, intelligent cardiotocography classification, fetal distress diagnosis, multimodal learning, vit, transformer

## Abstract

Cardiotocography (CTG) monitoring is an important medical diagnostic tool for fetal well-being evaluation in late pregnancy. In this regard, intelligent CTG classification based on Fetal Heart Rate (FHR) signals is a challenging research area that can assist obstetricians in making clinical decisions, thereby improving the efficiency and accuracy of pregnancy management. Most existing methods focus on one specific modality, that is, they only detect one type of modality and inevitably have limitations such as incomplete or redundant source domain feature extraction, and poor repeatability. This study focuses on modeling multimodal learning for Fetal Distress Diagnosis (FDD); however, exists three major challenges: unaligned multimodalities; failure to learn and fuse the causality and inclusion between multimodal biomedical data; modality sensitivity, that is, difficulty in implementing a task in the absence of modalities. To address these three issues, we propose a Multimodal Medical Information Fusion framework named MMIF, where the Category Constrained-Parallel ViT model (CCPViT) was first proposed to explore multimodal learning tasks and address the misalignment between multimodalities. Based on CCPViT, a cross-attention-based image-text joint component is introduced to establish a Multimodal Representation Alignment Network model (MRAN), explore the deep-level interactive representation between cross-modal data, and assist multimodal learning. Furthermore, we designed a simple-structured FDD test model based on the highly modal alignment MMIF, realizing task delegation from multimodal model training (image and text) to unimodal pathological diagnosis (image). Extensive experiments, including model parameter sensitivity analysis, cross-modal alignment assessment, and pathological diagnostic accuracy evaluation, were conducted to show our models’ superior performance and effectiveness.

## Introduction

Electronic fetal monitoring is a commonly used technique by obstetricians and gynecologists to assess fetal well-being during pregnancy as well as labor periods (Saleem and Naqvi, 2019). In this regard, cardiotocography (CTG) records changes in Fetal Heart Rate (FHR) signals and their temporal relationship with uterine contractions, which can be applied noninvasively and plays a critical role in Fetal Distress Diagnosis (FDD) (Santo et al., 2017). Among this, FHR can provide the main information about the relationship between sympathetic and parasympathetic nervous systems and their balance, and is an important parameter for the clinical evaluation of fetal well-welling (Black et al., 2004). Therefore, developing a high-precision Intelligent CTG (ICTG) classification model based on the FHR for prompt FDD is crucial for pregnancy management. The relevant literature has shown that the incidence of fetal distress in prenatal fetal monitoring is approximately 3%–39%, which further indicates the significance of antenatal fetal monitoring (Zhang and Yan, 2019).

Artificial Intelligence (AI) has witnessed many significant advances in the FDD community, where One-Dimensional (1D) original FHR signals (Fergus et al., 2021), various feature indicators (Hussain et al., 2022), and transformed Two-Dimensional (2D) images (Liu M. et al., 2021) are mainly used to explore the physiological and pathological information about pregnant women and fetuses. These methods have demonstrated great potential in accurately detecting fetal well-being, however, still have some challenges. For example, one major limitation of feature engineering is that it is subjective, and feature indicators depend on the experience of clinical experts and are not completely independent and objective, which has poor repeatability (Comert and Kocamaz, 2019; Fergus et al., 2021). Furthermore, unimodal input data or insufficient feature indicators is easy to cause incomplete source domain feature extraction. In contrast, too many feature indicators will bring about redundancy (Gao et al., 2013; Hu and Marc, 2018). Therefore, it is difficult to realize an accurate diagnosis in the clinic even with repeatedly trained and high-performance classifiers. Due to the complementarity among different modals, the joint representation can overcome the limitations of local features in the original signal or image feature representation (Kong et al., 2020; Rahate et al., 2022). This raises important questions: Can we integrate representations from these multimodalities to exploit their complementary advantages for FDD? To what extent should we process the different modalities independently, and what kind of fusion mechanism should be employed for maximum performance gain?

Data fusion is the combination of data from different modalities and sources that provide separate perspectives on a common phenomenon and is performed differently to predict a precise and proper outcome, which is also known as multimodal fusion (Tadas et al., 2018). This has the potential to solve problems with fewer errors than unimodal approaches would (Richard et al., 2022). In recent years, multimodal AI methods have been increasingly studied and used in various fields (Li et al., 2018; Baltrusaitis et al., 2019), and multimodal Deep Learning (DL) provides advantages over shallow methods for data fusion. Specifically, it can model nonlinearity and cross-modality relationships, which has expanded its range of applications from Computer Vision (CV) to Natural Language Processing (NLP) to the biomedical field (Ramachandram and Taylor, 2017; Bichindaritz et al., 2021). However, it faces specific challenges in biomedical applications, particularly as multimodal biomedical data typically have misaligned properties or labels, which raises the problem of studying more complex models and analyzing biomedical data.

Recently, Transformer-based multimodal fusion framework has been developed to address numerous typical issues using the multi-head attention mechanism. It is a typical encoder-decoder architecture that not only revolutionized the NLP field but also led to some pioneering work in the field of CV. By introducing the standard Transformer (Vaswani et al., 2017) and Vision Transformer (ViT) (Dosovitskiy et al., 2021) as the basis, Wang et al. (2020), Tsai et al. (2019), and Prakash et al. (2021) all proposed different variants to adapt streams from one modality to another, allowing us to explore the correlation between multimodal knowledge effectively. However, the acquisition of multimodal biomedical data is typically non-synchronous in clinical settings, particularly health data involving patients’ personal information and privacy, but these approaches require all modalities as input. As a result, they are rather sensitive and difficult to implement in the absence of modalities.

In this study, we focus on modeling multimodal learning for FDD. To solve the above problems in principle, we propose a Multimodal Medical Information Fusion (MMIF) framework that combines two backbones of the Category Constrained-Parallel ViT framework (CCPViT) and the Multimodal Representation Alignment Network (MRAN), allowing the modeling of both image- and text-based unimodal features and cross-modality fusion features. Compared with most existing FHR-based unimodal classification models, MMIF is an image-text foundation model that could contribute to a much higher-precision model. The main contributions of this study can be summarized as follows:

1) CCPViT is first proposed and used to learn key features of different modalities and solve the unaligned multimodal task. We use an image encoder to extract the encoding features of 2D images based on the Gramian Angular Field (GAF). Then, all labels are treated simply as unimodal text-only representations and decoded using a Unimodal Text Decoder to align the image features. Simultaneously, it is regarded as a constraint and controls the entire multimodal learning task.
2) MRAN is further proposed. It is a multimodal text decoder. There is a strong causality and inclusion between the above two modalities. We introduced cross-attention to establish an image-text joint component that cascades the encoded unimodal image features and the decoded unimodal text features to further explore the deep-level interactive representation between cross-modal data, thereby assisting the modal alignment, and further identifying abnormal behaviors.
3) Based on the learned MMIF, we designed a simple-structured FDD test model to enable it to satisfy downstream tasks and realize the FHR-based FDD task with an image-only modality as input. For evaluation, MMIF was verified on a public clinical database. The experiments demonstrate that MMIF can achieve state-of-the-art or even better performance than baseline models.

## Related work

In this section, a review of off-the-shelf ICTG methods based on the FHR, as well as basic information about multimodal fusion methods, is presented.

### FHR-based ICTG approaches

#### Computerized CTGs

They are mainly based on a programmatic calculation of authoritative guidelines here and abroad. To achieve consistent detection, several international authoritative guidelines, including SOGC (Moshe et al., 2015), FIGO (Black et al., 2004), and Chinese expert consensus (Yang et al., 2015), have proposed many evaluation indicators based on CTG. Then, numerous software were developed on the basis of these guidelines to perform CTG analysis automatically. 2CTG2 (Magenes et al., 2007), SisPorto (Ayres-de-Campos et al., 2017), CTG Analyzer (Sbrollini et al., 2017) and CTG-OAS (Comert and Kocamaz., 2018) are some of them. Since these software mostly uses the indicators inside guidelines as regulations, it causes high sensitivity and low specificity in practical applications, particularly when CTG cases are less than 40 min, false positives are more likely to occur, which will lead to excessive intervention.

#### Feature engineering-based ICTGs

These approaches focus on the analysis of basic feature engineering by mimicking the diagnosis of clinical obstetrics and gynecology experts and combining it with AI algorithms, thereby identifying the fetal status. Feature engineering primarily includes time-domain and frequency-domain feature engineering in this context. The former relates to morphological, linear, nonlinear, and high-order statistical features (Zhang Y. et al., 2019; Signorini et al., 2020; Chen et al., 2021), and the latter includes various classical frequency spectra (Zhang Y. et al., 2019; Zeng and Lu, 2021). For example, Zeng and Lu. (2021) explored CTG signal’s non-stationarity and class imbalance by adopting linear, time-domain and frequency-domain features for training an Ensemble Cost-sensitive Support Vector Machine (ECSVM) classifier. Hussain et al. (2022) proposed an AlexNet-SVM model to explore pathological information from numerous feature indicators of FHR signals. As seen from Table 1, both the studies of Chen et al. (2021) and Hussain et al. (2022) achieved good diagnostic accuracy, which is partly because their experimental database consists of numerous feature indicators calibrated by clinical experts. That is, they depend on the experience of clinical experts and are not completely independent and objective.

**TABLE 1:** A review and comparison of the existing ICTG classification models.

	Authors	Dateset (normal/pathological)	Algorithm	Performance
Feature engineering-based ICTG	Zhang Y. et al. (2019)	CTU-UHB (509/43)	LS-SVM + GA	ACC = 0.910, AUC = 0.920
	Signorini et al. (2020)	Private (60/60)	Random Forest	ACC = 0.911, Sen = 0.902
	Chen et al. (2021)	UCI (1655/176)	Deep Forest	ACC = 0.951, F1 = 0.920
	Zeng and Lu. (2021)	CTU-UHB (442/27)	ECSVM	Sen = 0.852, Spe = 0.661
	Hussain et al. (2022)	UCI (Total: 2126)	AlexNet-SVM	ACC = 0.993, Sen = 0.967
1D signal/2D image-based ICTG	Comert et al. (2017)	Private (272/44)	DWT + kNN + ANN	ACC = 0.905 (Normal)
				= 0.902 (Pathological)
	Comert and Kocamaz., (2019)	CTU-UHB	STFT + DCNN-TL	ACC = 0.933
	Fuentealba et al. (2019)	CTU-UHB (354 + 18)	CEEMD + SVM	ACC = 0.817
	Zhao et al. (2019)	CTU-UHB (447 + 105)	CWT + CNN	ACC = 0.983, AUC = 0.978
	Fergus et al. (2021)	CTU-UHB (506/46)	1D FHR + CNN	AUC = 0.860
	Liu M. et al. (2021)	CTU-UHB (439/113)	1D FHR + DWT + CNN-BiLSTM	ACC = 0.717, Sen = 0.752

Note: LS-SVM + GA: Genetic Algorithm and Least Square SVM; kNN: k-Nearest Neighbor; ANN: artificial neural network; TL: transfer learning; BiLSTM: Bidirectional Long Short-Term Memory.

#### 1D signal/2D image-based ICTGs

Contrary to complex feature engineering, original FHR signals can also be used as input directly to achieve the same purpose. By introducing the standard Convolutional Neural Network (CNN) as the basis, both Fergus et al. (2021) and Liu M. et al. (2021) achieved FDD by exploring pathological information from the original FHR. Meanwhile, various transformations, such as Continuous and Discrete Wavelet Transforms (CWT, DWT) (Comert et al., 2017; Zhao et al., 2019), Short Time Fourier Transform (STFT) (Comert and Kocamaz., 2019) and Complete Ensemble Empirical Mode Decomposition (CEEMD) (Fuentealba et al., 2019), have also been used in ICTG. These time–frequency-domain signal processing techniques are typically combined with DL models. Among these, Liu M. achieved ICTG by capturing pathological information with the combination of 1D FHR signals and DWT-based 2D images (Liu M. et al., 2021). Table 1 provides a detailed review of these FDD models. Compared with feature engineering, 1D signal/2D image-based ICTGs have improved in accuracy on some specific datasets. Therefore, the application of these algorithms in different clinical settings is worthy of further exploration.

### Multimodal fusion approaches

**FIGURE 1:**
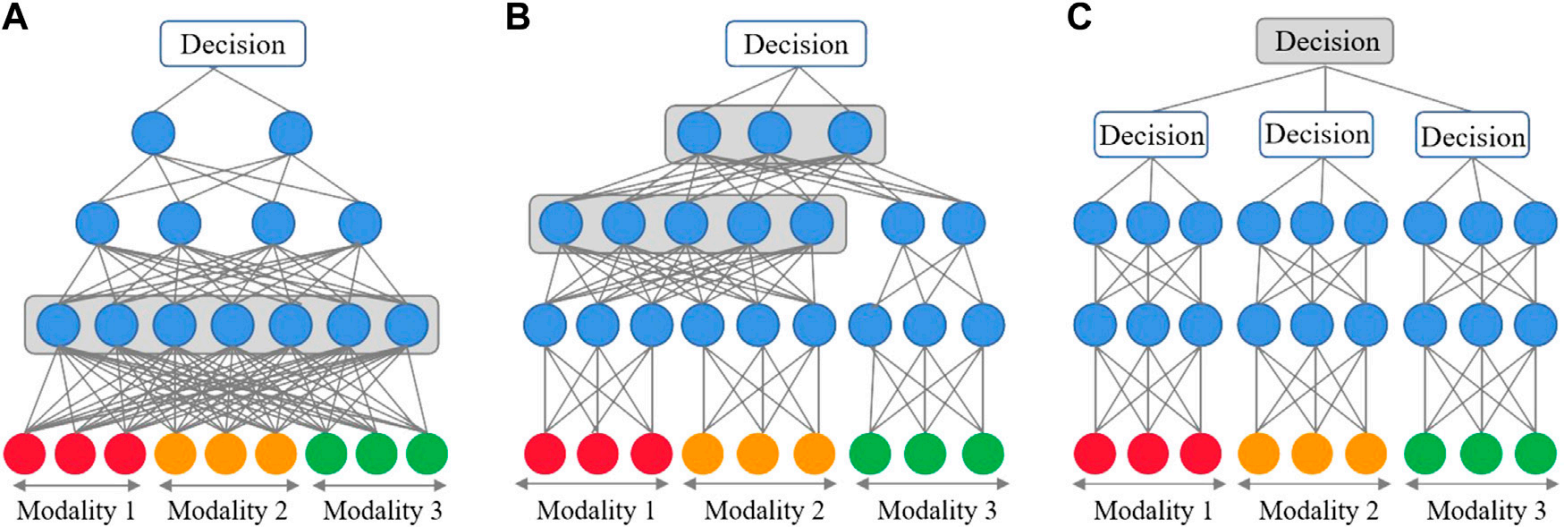
DL-based fusion strategies. Layers marked in red are shared between modalities and learned joint representations. **(A)** Early fusion takes concatenated vectors as input; **(B)** Intermediate fusion first learns marginal representations and fuses them later inside the network. This can occur in one layer or gradually. **(C)** Late fusion combines decisions by submodels for each modality.

Considering different fusion positions, conventional fusion is generally divided into early, intermediate, late, and hybrid fusion techniques (shown in Figure 1). Among those, early fusion is also known as feature-based fusion, which focuses on learning cross-modal relationships from low-level features (Mello and Kory, 2015). In intermediate fusion, marginal representations as feature vectors are learned and fused instead of the original multimodal data (Lee and Mihaela, 2021). In contrast, late fusion performs integration at the decision level by voting among the model results; thus, it is also known as decision-based fusion (Torres et al., 2022). In hybrid fusion, the output comes from a combination of the first three fusion strategies (Tsai et al., 2020). Inspired by the success of the Transformer model, the standard Transformer and ViT structures, as well as various variants based on them, have been widely used in multimodal data learning (Tsai et al., 2019; Wang et al., 2020; Prakash et al., 2021). ViT has achieved excellent performance on multiple benchmarks, such as ImageNet, COCO, and ADE20k, compared to CNNs.

## Methodology

**FIGURE 2:**
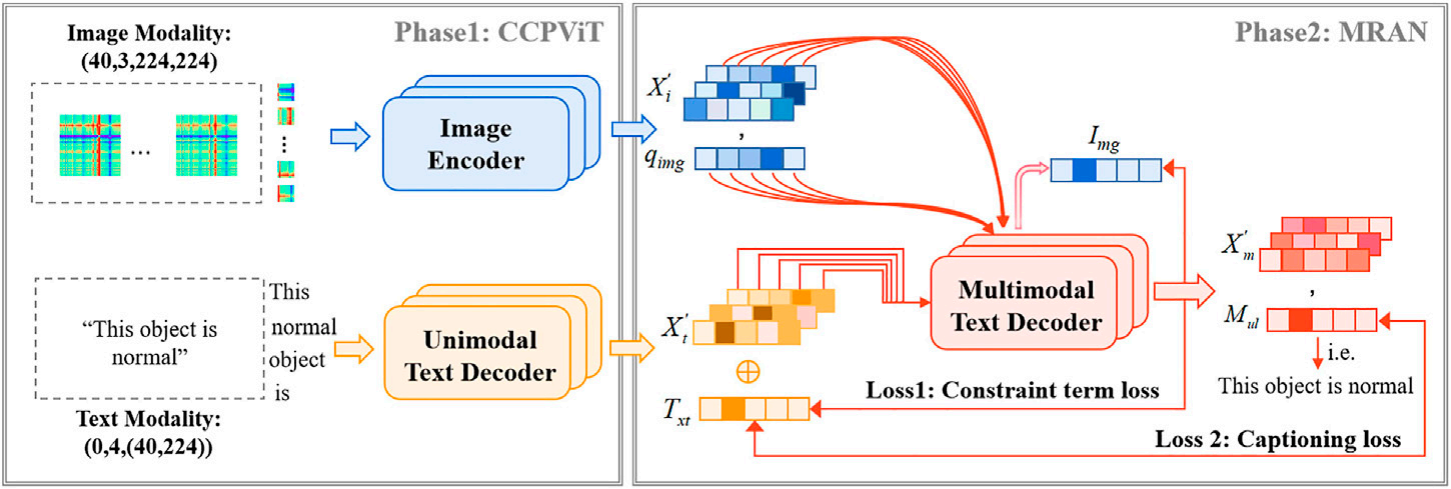
Detailed illustration of MMIF during the training period. CCPViT is utilized to learn the unimodal text and image features. MRAN is applied to explore the deep-level interactive representation between cross-modal data. Multimodal fusing embeddings are input to the MMIF model to generate text-only representations.

**FIGURE 3:**
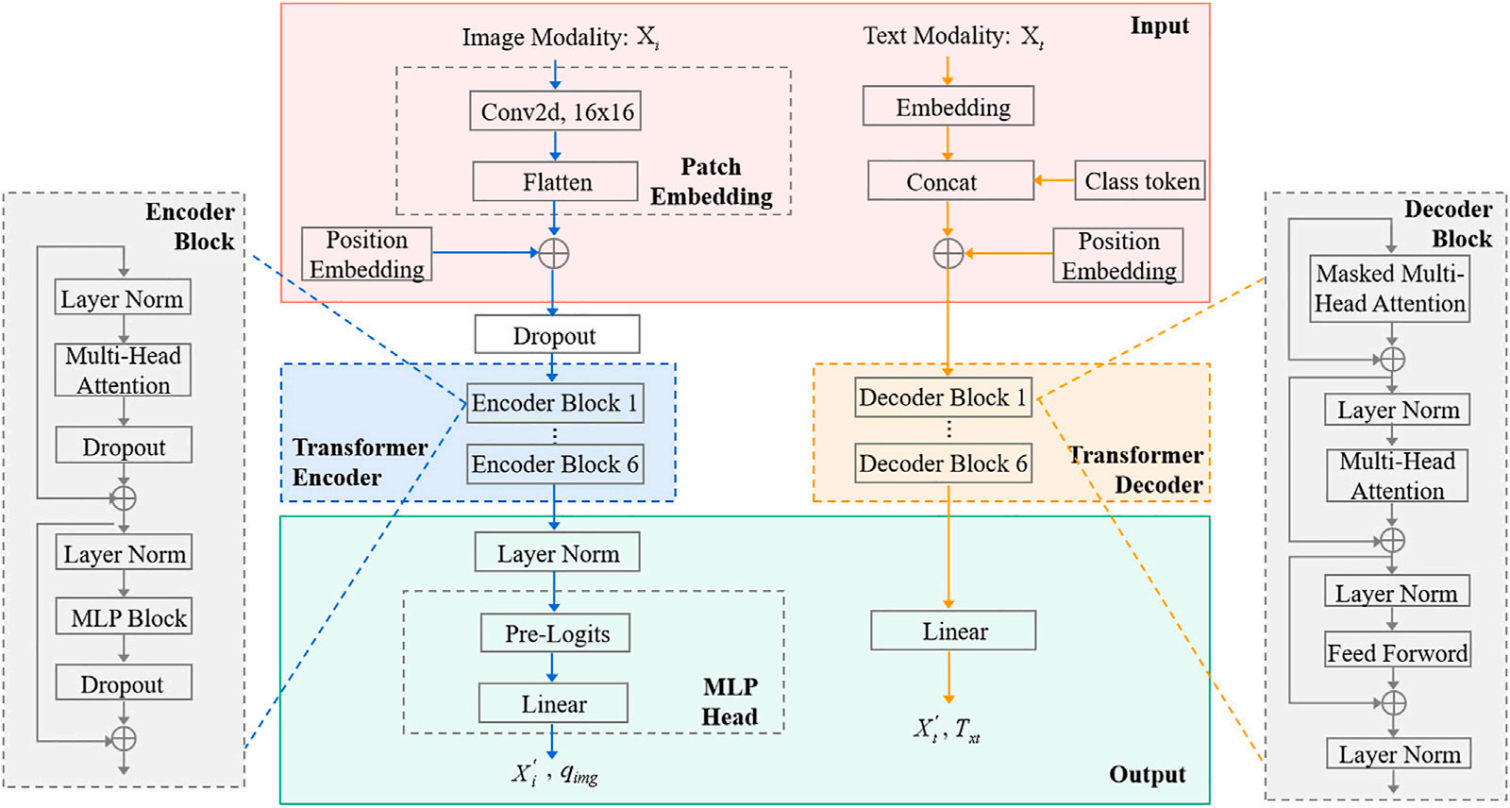
CCPViT: Xi and Xt refer to the features of image modality and text modality, respectively. The input image is a 224×224 RGB image. The backbones of the image encoder and unimodal text decoder are shown in the blue and yellow boxes, respectively.

**FIGURE 4:**
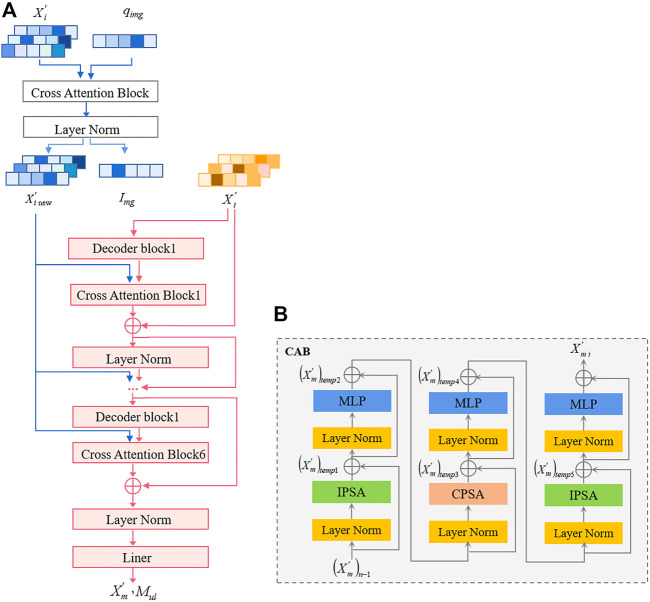
MRAN: The input data are the outcome of CCPViT, i.e., the encoded unimodal image features $X_{i}^{\prime }$ and its image query $q_{img}$ , and the decoded unimodal text features $X_{t}^{\prime }$ and its [class_text] token $T_{xt}$ . The multimodal text decoder is used to exploit the local and explicit interplay between cross-modality translations. **(A)** The network structure of MRAN; **(B)** The network structure of CAB.

**FIGURE 5:**
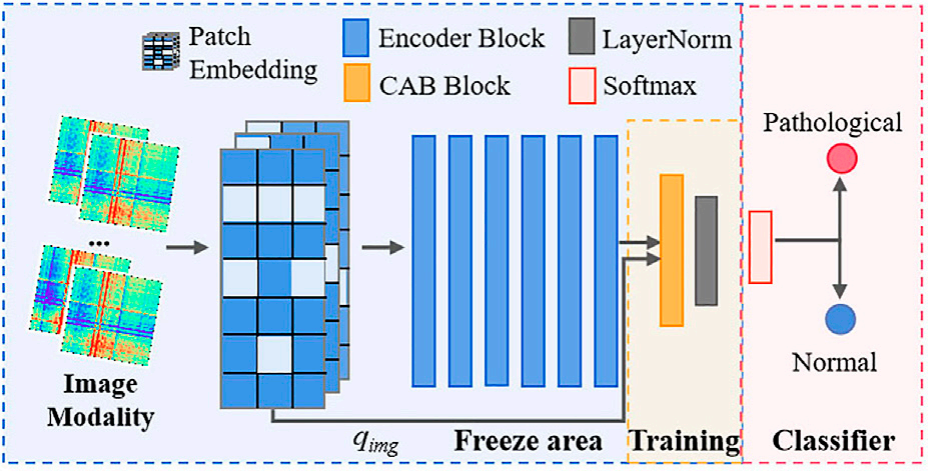
Application of the trained MMIF to the FDD task. MMIF is composed of a frozen Transformer Encoder and a CAB structure for aggregating features and producing pathological diagnosis results.

In this section, we first present the input representation, which is a simple image and text modality. Then, we elaborated on the MMIF framework (Figure 2), which includes CCPViT (Figure 3) and MRAN (Figure 4) Finally, we presented an FDD test model (Figure 5), which is used to satisfy the constraints of data from different source domains in clinical practice.

### Preliminaries

Given the length of an original FHR signal as L, we use $X_{s}\in R^{1\times L}$ to represent a series of collected FHR signals. The inputs of the backbone consist of two modalities, image ( I ) and text ( T ), and can be denoted as $X_{i}\in R^{n_{i}\times d_{i}}$ and $X_{t}\in R^{n_{t}\times d_{t}}$ , respectively. Here, $n_{i}$ and $n_{t}$ represent the number of tokens, i.e., image and word numbers, respectively. $d_{i}$ represents the image size, and $d_{t}$ represents the dimension of text features. Then, the output feature representations of I and T are denoted as $X_{i}^{\prime }$ and $X_{t}^{\prime }$ , i.e., [img_embeds] and [text_embeds], respectively. $I_{mgi}$ and $T_{xtj}$ denote the outcome label of the *i*th pair of image and text modalities, respectively, i.e., [class_img] and [class_text] tokens. Meanwhile, this study denotes image–text multimodality as M ; thus, the outputs of M can be denoted as $X_{m}^{\prime }$ (cross-modality fusion features, i.e., [mul_embeds]) and $M_{uli}$ (the *i*th pair of multimodal prediction results, i.e., [class_mul] tokens).

#### Multi-head self-attention mechanism

This study follows the standard mechanism of multi-head self-attention. For simplicity, we consider the unimodality representation $X_{i}\in R^{n_{i}\times d_{i}}$ as an example of the translation process. First, $X_{t}\in R^{n_{t}\times d_{t}}$ is delivered to a densely connected layer for linear projection to obtain the updated $X_{i}^{\prime }\in R^{n_{i}\times L_{i}}$ , where $L_{i}$ represents the output dimension of the linear layer. Thus, the corresponding query matrix, key matrix and value matrix are denoted as $Q_{i}=X_{i}W^{Q_{i}}\in R^{n_{i}\times L_{i}}$ , $K_{i}=X_{i}W^{K_{i}}\in R^{n_{i}\times L_{i}}$ , and $V_{i}=X_{i}W^{V_{i}}\in R^{n_{i}\times L_{i}}$ , where $W^{Q_{i}}$ , $W^{K_{i}}$ , and $W^{V_{i}}$ represents weight matrices. Then, we compute the scaled dot products between $Q_{i}$ and $K_{i}$ , divide each by the scale coefficient $\sqrt{L_{i}}$ , and use a softmax function to obtain the attention weights with $V_{i}$ .

$$head=Attention\left(Q_{i},\;K_{i},\;V_{i}\right)=soft\max\left(\frac{Q_{i}K_{i}^{T}}{\sqrt{L_{i}}}\right)V_{i}$$ (1)

Note that the Transformer applies the attention mechanism several times throughout the architecture, resulting in multiple attention layers, with each layer in a standard transformer having multiple parallel attention heads:

$$Multi-head=Concat\left({head}_{1}{,\;...,\;head}_{h}\right)W^{O}$$ (2)

### Input representation

#### Image modality-2D images based on Gramian Angular Difference Fields (GADF)

GAF is a time series encoding method based on the inner product and the Gram matrix proposed by Zhiguang Wang and Tim Oates in 2015 that allows each group of FHR series to generate only one polar coordinate system-based mapping map (Wang and Oates, 2015). First, the FHR is scaled in the Cartesian coordinate system to [−1,1] . Then, it is converted into a polar coordinate system time series. Specifically, take the time axis as the radius and the FHR value as the cosine angle. Finally, GADF images can be obtained by angle difference-based trigonometric function transformation (Eq. 3). Because the sequence length will extensively affect the calculation efficiency, we introduced the piecewise aggregation approximation to obtain the dimension value. Experimentally, set the initial dimension as L=7200 (the original FHR length) and the fixed difference as 180, then decrease the dimension from 7200 to 180 in turn, and a total of 40 sets of parameters can be obtained, i.e., [7200,7020,...,180] . Thus, 40 GADF-based 2D images with an image size of 224 × 224 are obtained, each of which can be marked as $X_{i}\in R^{40\times 224}$ .

$$GADF=\left[\sin\left(\varphi_{i}-\varphi_{j}\right)\right]={\sqrt{I-{\tilde{X}}_{s}^{2}}}^{\prime }\cdot {\tilde{X}}_{s}-{\tilde{X}}_{s}^{\prime }\cdot \sqrt{I-{\tilde{X}}_{s}^{2}}$$ (3)

where I represents a standard row vector, and ${\tilde{X}}_{s}$ is the scaled time series.

#### Text modality-description of pathological diagnosis

Sample labels were introduced to construct unimodal text-only data and used as a constraint of MMIF. For the training and validation sets with known pathological status, the description criteria adopted are as follows: if the current sample is normal, obtain its text modality as “This object is normal”; otherwise, an abnormal FHR can be described as “This object is pathological”. Set the model dimension to 224; thus, the text modality can be quantized as $X_{i}\in R^{4\times 224}$ .

### The MMIF model

The goal of this work is to achieve a high-precision intelligent FDD. To achieve this, an MMIF framework has been elaborately devised, as shown in Figure 2. It mainly consists of two backbones: CCPViT and MRAN. The former is applied to feature encoding in the case of unaligned multimodalities, and the latter is capable of exploring the deep-level interactive representation among cross-modal data and further assisting modal alignment.

#### Category constrained-parallel ViT

The backbone of CCPViT is a simple encoder–decoder architecture with multiple standard ViT Encoders and Transformer Decoders, as shown in Figure 3. Our key idea is to exploit the multi-head self-attention mechanism to model from two unimodality representations, $X_{i}$ and $X_{t}$ .

First, we focused on learning the encoding features of GADF images and constructed an image encoder with ViT-B/16 as the backbone. Inspired by the success of the Transformer in NLP, ViT was originally developed by Google Research in 2020 to apply the Transformer to image classification (Dosovitskiy et al., 2021). ViT-B/16 is the model variant of the standard ViT, which means the Base variant with an input patch size of 16 × 16. Compared with the traditional convolution architecture, the most significant advantage of ViT-B/16 is that it has a great vision in both shallow and deep structures, which ensures that it can not only obtain global feature information in the shallow layer but can also learn high-quality intermediate features in the middle layer and retain more comprehensive spatial information in the deep layer, resulting in excellent classification performance. In this study, our proposed image encoder consists of three stages: In the input stage, we split an image into patches and sequentially sort them to form a linear embedding sequence named patch embeddings, which are then fed to the Transformer to realize the application of the standard Transformer in CV tasks. Meanwhile, position embeddings were added to remember the positional relationship between these patches. In the second stage, the Transformer Encoder block was used as the basic network module, with each primarily including LayerNorm, multi-head attention, dropout, and MLP Block structure. We repeatedly stacked it six times to continuously deepen the network structure and mine typical features of the input data. A standard MLP head was introduced in the final stage to output the encoded unimodal image features $X_{i}^{\prime }$ and a learnable image query $q_{img}\in R^{1\times d_{i}}$ from query matrix $Q_{i}$ . It is worth noting that $q_{img}$ was mainly used for cross-modal learning in MRAN.

Subsequently, we studied the sample label-based unimodal text data. Specifically, an independent unimodal text decoder was established in this study that does not interact with the image side’s information. It uses the Transformer Decoder as the decoding block, which is an efficient and adaptive method for retrieving the long-range interplay along the temporal domain. Similarly, we divide the unimodal text decoder into three stages. The first is a standard embedding layer to obtain text tokens. Then, the decoder block is stacked by six decoders, each of which is composed of masked multi-head attention, multi-head attention, and a feed-forward network structure. Finally, we can obtain the decoded unimodal text features $X_{t}^{\prime }$ and its [class_text] token $T_{xt}$ with a linear transformation structure.

Note that there is no cross-attention between the Unimodal Text Decoder and Image Encoder, which are two parallel feature representation models. Hence, it is difficult to align the decoded text features with the global image information. This study addresses this misalignment from two aspects: On the one hand, similar to ALBEF’s [class] token (Li and Selvaraju, 2021), we inserted a [class_text] token into the patch embeddings in the unimodal text decoder, which combines a constraint term loss with the encoded features on the image side for comparison learning. Note that the [class_text] token in this stage is a trainable parameter, whose output state at the third stage serves as the outcome label of the text modality; Another auxiliary alignment is primarily reflected in MRAN, which will be specified in the following part.

#### Multimodal representation alignment network

The backbone of MRAN is a multimodal text decoder, which is located above the image encoder and unimodal text decoder. It cascades with the output of image encoding through a cross-attention network to learn the multimodal image–text representation, generates interactive information about them, and then decodes the information to restore the corresponding text, that is, the text-only representation of the pathological diagnosis results.

An overview of the backbone architecture is shown in Figure 4. The input of MRAN is the output of CCPViT, including the encoded unimodal image features $X_{i}^{\prime }$ and its image query $q_{img}$ , the decoded unimodal text features $X_{t}^{\prime }$ and its [class_text] token $T_{xt}$ . The entire process of MRAN consists of three stages: Stage 1, update the information on the image side. $X_{i}^{\prime }$ and $q_{img}$ are input into a Cross-Attention Block (CAB) structure to capture local information, which is then normalized through a LayerNorm layer to smooth the size relationship between different samples and retain it between different features. Therefore, the updated information on the image side was obtained, and then the updated image features $X_{inew}^{\prime }$ (columns 1st to $\left(d_{i}-1\right)th$ ) and its [class_img] token $I_{mg}$ (the last column) can be obtained. Stage 2, cross-modal learning. The structure of the multimodal text decoder primarily includes decoder blocks and CAB modules. In this stage, $X_{t}^{\prime }$ first went through the decoder block once and then was cascaded with $X_{inew}^{\prime }$ .Next, they were jointly input into a CAB structure for cross-modal learning. Repeatedly stack six times and combine constraint term loss and captioning loss to evaluate the performance of cross-modal learning to achieve a deep-level of interaction and optimization. Stage 3, standardized processing and output the joint representation of image–text information $X_{m}^{\prime }$ and the text-only representation of pathological diagnosis results $M_{ul}$ .

The structure and calculation process of the CAB are shown in Figure 4B, which primarily includes two Inner Patch Self-Attentions (IPSAs) and one Cross Patch Self-Attention (CPSA). It is a new attention mechanism that does not calculate the global attention directly but controls the attention calculation inside the patch to capture local information and then applies attention to the patches between single-channel feature maps to capture global information. Based on the CAB, a stronger backbone can be constructed to explore the causality and inclusion between image and text modalities and generate multiscale feature maps, satisfying the requirements of downstream tasks for features with different dimensions.

#### Modeling alignment of cross-modal

Throughout the training process of MMIF, decoded unimodal text features were combined with encoded unimodal image features for multimodal learning and pathological diagnosis. The text modality acts as a hard constraint to restrict the entire cross-modal learning. Specifically, we divided the modal alignment task into two parts: unimodal alignment between image and text labels, and multimodal alignment between multimodal and text labels, and defined two indicators to measure their alignment degree.

1) Constraint term loss: This is the training loss of CCPViT, marked as $L_{con}$ . We use an exponential loss function to comparatively learn the difference between $T_{xt}$ and $I_{mg}$ , as shown in Eqs 4–6. Notably, the contrastive learning from I to T and T to I are marked as ${Trans}_{I\to T}$ and ${Trans}_{T\to I}$ , respectively; thus, $L_{I\to T}$ and $L_{T\to I}$ denote the training losses of ${Trans}_{I\to T}$ and ${Trans}_{T\to I}$ , respectively.

$$L_{I\to T}=-\frac{1}{N}\underset{i}{\sum }\log\left(\frac{\exp\left(\frac{I_{mgi}^{T}\cdot T_{xti}}{\tau }\right)}{\sum_{j}^{N}\exp\left(\frac{I_{mgi}^{T}\cdot T_{xtj}}{\tau }\right)}\right)$$ (4)

$$L_{T\to I}=-\frac{1}{N}\underset{i}{\sum }\log\left(\frac{\exp\left(\frac{T_{xti}^{T}\cdot I_{mgi}}{\tau }\right)}{\sum_{j}^{N}\exp\left(\frac{T_{xti}^{T}\cdot I_{mgj}}{\tau }\right)}\right)$$ (5)

$$L_{con}=L_{I\to T}+L_{T\to I}$$ (6)

where $I_{mgi}$ and $T_{xtj}$ denote the outcome label of the *i*th pair of image modalities and the *j*th pair of text modalities. N refers to the batch size, and τ is the temperature to scale the logits.

2) Captioning loss: This is the training loss of MRAN, marked as $L_{cap}$ . It calculates the cross-entropy loss of $T_{xt}$ and $M_{ul}$ , assisting the alignment of multimodal data.

$$L_{cap}=\frac{1}{N}\underset{i}{\sum }l_{i}=\left\{ \begin{array}{l} -\underset{i}{\sum }\overset{C}{\underset{c=1}{\sum }}M_{ul\;ic}\cdot \log\left(p_{ic}\right),if\;multi\;classification\\ -\frac{1}{N}\underset{i}{\sum }\left[M_{ul\;i}\cdot \log\left(p_{i}\right)+\left(1-M_{ul\;i}\right)\cdot \log\left(1-p_{i}\right)\right],if\;binary\;classification \end{array}\right.$$ (7)

where $M_{uli}$ denotes the label of the *i*th multimodal prediction results, the positive (i.e., normal) and negative (i.e., pathological) FHRs are labeled as 0 and 1, respectively; and $p_{i}$ denotes the probability that the *i*th multimodal sample is classified as normal.

Therefore, the calculation of the loss function throughout the training process can be obtained (Eq. 8), where α and β refer to the loss coefficient weights of constraint term loss and captioning loss, respectively, and α+β=1 . In subsequent experiments, α and β are important hyperparameters to be optimized.

$$L=\alpha \cdot L_{con}+\beta \cdot L_{cap}$$ (8)

### MMIF for FDD task

In clinical practice, the acquisition of multimodal biomedical data is typically non-synchronous, especially health data containing patients’ personal information and privacy. Thus, an optimal solution is to develop a diagnostic model to satisfy the constraints of data from different source domains in clinical tasks. Based on the goal of this study, we cannot obtain the text modality in advance in actual clinical diagnosis. In contrast, obtaining pathological diagnosis results (i.e., text modality) is our eventual goal. Therefore, we designed an FDD test model shown in Figure 5 to implement the FHR-based FDD task, using image-only modalities as input.

Specifically, we first convert the 1D FHR signal of the object being diagnosed to GADF-based 2D images and feed each image into the trained image encoder separately. For the trained image encoder, freeze its feature layers, i.e., freeze the last layer of the 6th Transformer Encoder block and that before the MLP head. Then, cascade a CAB with a LayerNorm layer on top of the feature tokens and its single image query token, then feed to a softmax cross-entropy loss, thereby completing the FDD task applicable to image-only modalities and realizing effective diagnosis of fetal health status.

In principle, the fundamental reason why the proposed MMIF can be adapted to different types of downstream tasks (that is, with different modalities as input data) by sharing the backbone encoder is that it is a parallel model in which the unimodal text decoder and image encoder are independent of each other, and the trained model is highly modal alignment. The powerful performance of the trained encoder is based on the joint learning of local-level text and global image modalities.

## Experiments and discussion

In this section, we extensively evaluate the capabilities of MMIF as a trained model in downstream tasks. We focus on three categories of tasks: 1) parameter sensitivity analysis, 2) cross-modal alignment of image, text and multimodal understanding capabilities, and 3) pathological diagnosis of the FDD test model. Since MMIF achieves both unimodal representations and fused multimodal embeddings simultaneously, it is easily transferable to all three tasks. The results verified that the proposed MMIF achieves state-of-the-art diagnosis accuracy (0.963).

### Experimental setup

#### Datasets

A publicly available intrapartum CTG dataset, which is available on Physionet (Goldberger and Amaral, 2000), was used in this study. It initially contained 552 recordings collected from the obstetrics ward of the Czech Technical University-University Hospital in Brno (CTU-UHB), Czech Republic, between April 2010 and August 2012 (Václav et al., 2014). Clinically, the pH value of the neonatal umbilical artery and Apgar score at 5 and 10 min (Apgar 5/10) are gold standards to assess fetal health (Romagnoli and Sbrollini, 2020). The inclusion and exclusion criteria were as follows: 1) Rejected unqualified signals: The degree of the missing sample was greater than 10%. The missing beats increase during this period, and it is difficult to assess the FHR with increasing irregularity. 2) Signal length: L = 7200. Since the major fetal distress occurs before delivery, we focus on the last 30 min of the samples in the experiment (sampling frequency: 4 Hz). 3) Data partitioning: Normal FHR: pH≥7.15 and Apgar 5/10∈[9,10] ; Abnormal/pathological FHR: pH<7.05 . 4) Label the sample: label the normal and pathological FHRs as 0 and 1, respectively. According to this criterion, we collected a total of 40 pathological and 386 normal samples, from which 80 normal and 40 pathological samples were randomly selected, respectively.

#### Data preprocessing

The selected FHR signals were filled in using the mini-batch-based minimized sparse dictionary learning method (Zhang Y. et al., 2022). Subsequently, 40 pathological samples were augmented using the category constraint-based Wasserstein GAN model with gradient penalty to generate 40 simulated pathological signals, totaling 80 pathological samples. It is a small sample generation technology that is used to balance samples between positive and negative categories, as proposed in our previous study (Zhang Y.F. et al., 2022). Therefore, the structured database includes 80 normal and 80 pathological samples. Stratified random sampling was used to divide the samples into training and test sets in a 1:1 ratio. Then in the training process, 5-fold Cross Validation (5-CV) was used to further divide the training and validation sets. Specifically, since each sample contains 40 pairs of images and text data, 640 and 2560 data were used as validation and training sets respectively in each model training session.

#### Baseline methods

To demonstrate the effectiveness of our proposed MMIF, we compared it to two types of baseline methods, namely, feature engineering-based ICTGs and 1D signal/2D image-based ICTGs.

· LS-SVM + GA (Zhang Y. et al., 2019): Belongs to the first type. It combines a genetic algorithm and least square SVM for FDD, where 67 time–frequency-domain and nonlinear features are extracted.
· LocalCNN (Zhao et al., 2019): Belongs to the second type. It is a simple CNN model with an 8-layer CNN network, with 2D images obtained with the CWT as input data.
· VGGNet16: Belongs to the second type. It is a deep CNN model that uses VGGNet16 as a backbone and GADF-based 2D images as input data.
· ViT (Dosovitskiy et al., 2021): Belongs to the second type. Different from the above two traditional CNN architectures, we introduced ViT-B/16 for FDD, which uses GADF-based 2D images as unimodal input data.

#### Evaluation metrics

In our experiments, accuracy (ACC), F1-score (F1) and Area Under the Curve (AUC) were used as evaluation metrics. ACC measures how many objects were correctly classified. F1 balances the traditional precision and recall. AUC interprets the authenticity of the algorithm. Higher values of these three indicators indicate better link prediction performance. Furthermore, the coefficient of determination R-square (*R* ^2^), as shown in Eq. 9, was introduced to measure the ability of cross-modal alignment.

$$R^{2}=1-\frac{\overset{N}{\underset{i=1}{\sum }}{\left({\hat{y}}_{i}^{*}-T_{xti}\right)}^{2}}{\overset{N}{\underset{i=1}{\sum }}{\left(T_{xti}-{\hat{T}}_{xt}\right)}^{2}}$$ (9)

where ${\hat{y}}^{*}$ represents the outcome label of the image modality or multimodal prediction results, and ${\hat{T}}_{xt}$ denotes the mean of the outcome label of all text modalities.

#### Basic parameter settings

In the training process, set the batch size and the epoch to 32 and 10, respectively, and all training samples are trained on the combined loss function in Eq. 9. The loss is then backpropagated to update the parameters using AdaGrad, with exponential attenuation rates $\beta_{1}=0.9$ and $\beta_{2}=0.999$ and a decoupled weight decay rate of ${1e}^{-4}$ . The learning rate α is set to ${1e}^{-3}$ . In addition, the best value of temperature τ is empirically set as 0.07. For optimal accuracy, the two weight parameters α and β were set according to the model training results. In addition, the model structure was as follows: image encoder: depth is 6, and the number of multi-heads is 16; unimodal text decoder and MRAN: depth is 6, and the numbers of multi-heads is 8.

### Parameter sensitivity analysis

In this part, we focus on the effect of different weight parameters and decoder/encoder layers on the performance of MMIF to obtain an optimal model parameter combination.

**FIGURE 6:**
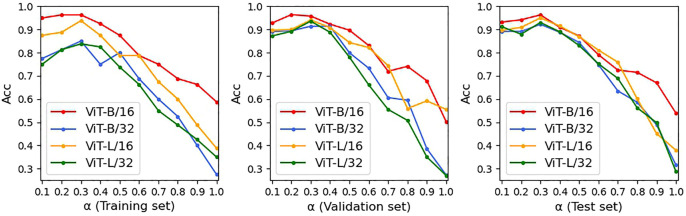
ACC score of link prediction on four different ViT structures, where red, blue, orange, and green refer to ViT-B/16, ViT-B/32, ViT-L/16, and ViT-L/32, respectively (set all blocks to six).

**FIGURE 7:**
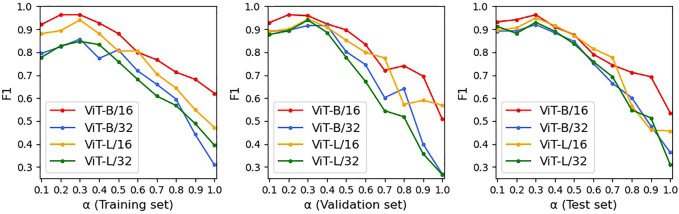
F1 score of link prediction on four different ViT structures, where red, blue, orange, and green refer to ViT-B/16, ViT-B/32, ViT-L/16, and ViT-L/32, respectively (set all blocks to six).

First, we analyzed the combination of α and β with the training and test sets in Figures 6, 7. The higher α is, the more important the constraint term loss. That is, MMIF focuses more on unimodal image encoding, and the performance of the image encoder plays a more important role in FDD diagnosis. Otherwise, MMIF pays more attention to multimodal image–text decoding. These figures show the changes in diagnostic ACC and F1 with various α . In this scope, we experimented with different ViT structures of CCPViT. As the weight of the constraint term loss ( α ) decreased, the prediction ability of each ViT model improved to varying degrees; however, when it was reduced below 0.3, the training performance of the four model structures was no longer improved, but the ViT-B/16-based model could still maintain high performance on the validation and test sets. The other three model structures, by contrast, show a significantly larger downward trend on both the validation and test sets. Thus, it is valid to consider that ViT-B/16 shows the most stable increase and the highest classification ACC and F1 in the FDD task. As shown in Figures 6, 7, ACC and F1 are maximized when the weight of the constraint term loss is set to α=0.3 and the weight of the captioning loss is β=0.7 .

**FIGURE 8:**
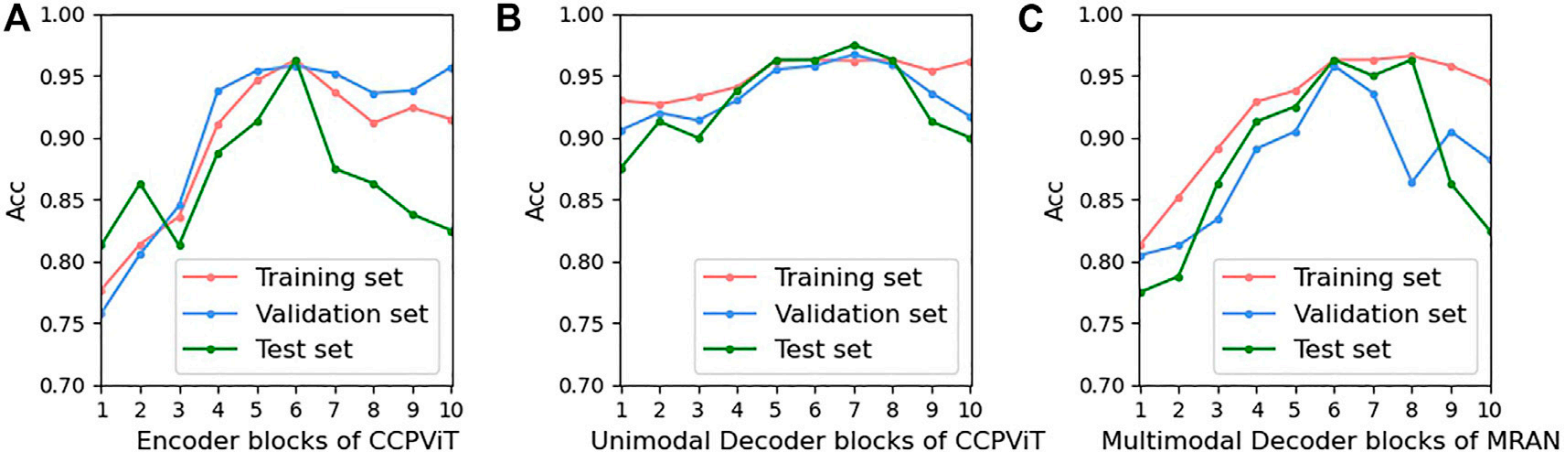
Effects of the encoder and decoder blocks on model prediction. The red, green, and blue curves refer to the training, validation, and test sets, respectively. Multimodal decoder blocks contain multi-head attention and CAB (select ViT-B/16 and set α=0.3 ; β=0.7 ). **(A)** Encoder blocks of CCPViT; **(B)** Unimodal Decoder blocks of CCPViT; **(C)** Multimodal Decoder blocks of MRAN.

We then tested the effect of different numbers of encoder and decoder blocks on the performance of the multimodal model, as shown in subgraphs (a)-(c) in Figure 8. Since each CCPViT and MRAN comprises several sequential encoder blocks and/or decoder blocks, we are skeptical that the final feature representation of a specific layer may affect the performance of the proposed model. According to the curves, as each network structure increases, the diagnostic accuracy of MMIF is significantly improved. In sub-graph (a), it is interesting to note that the model reaches the peak value at layer 6 on both the training and test sets, which means that the output of the 6th layer embraces the most discriminative fusion message. In comparison, the model is relatively less affected by unimodal decoder layers, which may imply that the lower layer can capture the joint representation for the simple case. The curve of sub-graph (c) shows a similar trend to that of (a) and achieves the optimal results at the 6th block of MRAN. In conclusion, the lower encoder and multimodal decoder blocks may involve low-level characteristics of interplay, whereas the higher encoder and multimodal decoder layers may embrace explicit messages. Comparing image-text modalities, text modality is the relatively simple case; thus, the lower unimodal decoder layer may be sufficient to demonstrate the interaction. By studying Figures 6–8, we can see that:

- ViT-B/16 shows the most stable increase and the highest classification ACC and F1.
- There is a sweet spot for the value of loss coefficient weights not significantly affected by ViT structures; thus, set α=0.3 and β=0.7 .
- There is a point of optimal balance in the encoder and decoder blocks; thus, set the encoder block in CCPViT, unimodal decoder in CCPViT, and multimodal decoder in MRAN to 6.

### Performance of cross-modal alignment

Cross-modal alignment is particularly important in multimodal learning. In the proposed MMIF, although both loss values are computed for the same final task, there is no explicit constraint being imposed on the consistency between their outcomes. Therefore, we must provide effective evidence and evaluation of the multimodal alignment of multimodal models.

In this part, we first examine the category output of two independent networks in the training set, that is, the output label of unimodal encoder $I_{mg}$ and multimodal decoder $M_{ul}$ , taking the category of unimodal decoder $T_{xt}$ as a contrast constraint. The results are listed in Table 2. First, the alignment of normal samples is slightly better than those of pathological cases in both scenarios of I→T and M→T . One possible explanation is that the main clinical manifestations of normal samples are highly similar and perform stably. Second, the value of $R^{2}\left(I\to T\right)$ (the alignment between $I_{mg}$ and $T_{xt}$ ) is slightly lower than that of $R^{2}\left(M\to T\right)$ (the alignment between $M_{ul}$ and $T_{xt}$ ), which, to some extent, proves that multimodal data fusion can reduce the shortcomings of local features in original signals or image feature representation to achieve a high-precision diagnosis. Finally, a high *R* ^2^ is achieved for both the unimodal encoder and multimodal decoder. Therefore, we believe that the diagnosis of our MMIF is sufficient to achieve high multimodal alignment attributes and is comparable to that of humans.

**TABLE 2:** Training result of cross-modal alignment (*R* ^2^).

		Normal samples	Pathological cases	The entire set
Training set	I→T	0.950 (0.939, 0.961)	0.935 (0.910, 0.959)	0.943
	M→T	0.967 (0.954, 0.980)	0.955 (0.941, 0.969)	**0.961**
Test set	-	0.968 (0.959, 0.978)	0.961 (0.944, 0.978)	**0.965**

Notes: I→T and M→T denote the alignment of encoded unimodal image features and text labels and the alignment of multimodal decoding features and text labels, respectively. The bold values means the best performance of the current experiments.

We then compared the alignment between the outcome of the FDD test model and its label category, as shown in the last row of Table 2. It can be found that it performs on par with MMIF on both normal samples and pathological cases. This finding suggests that the trained FDD classification model subsumes a strong learning property of MMIF. The effect of a softmax cross-entropy loss is equal to that of the two losses of MMIF when we use image-only modalities as input. Thus, our proposed MMIF can be interpreted as an effective unification of the three paradigms. This explains why the FDD test model in Figure 5 does not need a pretrained text decoder to perform well.

### Performance of pathological diagnosis

Over the years, several studies on FHR-based ICTG approaches have been conducted. To perform a more objective and comparative performance evaluation, we reproduced four baseline methods proposed in *Experimental Setup Section* and made a comparison with three evaluation metrics.

As shown in Table 3, our method has the best performance among all baseline methods. In particular, in terms of diagnostic ACC, MMIF-1 exceeds the previous best ViT method by a margin of 5%. One possible explanation is that MMIF performs well in the process of information interaction and feature learning for cross-modal data, which partly verifies the necessity of having a multimodal approach. Furthermore, in terms of F1, the empirical improvement of MMIF-1 is up to 8.4%. It is interesting to note that the improvement of DL methods, whether the CNN structure or models with ViT as a backbone, is more significant than LS-SVM + GA, a topical 1D feature engineering-based ICTG model. This implies that the 1D signal/2D image-based ICTG method is capable of improving the accuracy of pathological feature extraction, and furthermore, MMIF-1 can effectively utilize auxiliary features (text modality) to achieve deep-level interactive representations of data and self-learning of pathological features. The AUC is highly consistent with the other two indicators. We may reasonably conclude that although ICTG is a challenging task, DL-based diagnosis schemes are effective and our method is correct.

**TABLE 3:** Test results of our methods and baseline methods for FDD pathological diagnosis analysis.

Method	ACC	F1	AUC
LS-SVM + GA	0.863	0.879	0.863
LocalCNN	0.888	0.894	0.887
VGGNet16	0.900	0.894	0.900
ViT	0.913	0.916	0.912
MMIF-1 (Ours, ViT-B/16)	0.963	0.963	0.962
MMIF-2 (Ours, ViT-B/32)	0.850	0.857	0.850
MMIF-3 (Ours, ViT-L/16)	0.938	0.940	0.937
MMIF-4 (Ours, ViT-L/32)	0.850	0.860	0.847

Notes: The experiment is based on 40 normal and 40 pathological samples in the test set. The FDD, test model only employs unimodality (GADF-based 2D images) to perform the multimodal fusion task, as shown in Figure 5. The criteria to output the diagnostic classification result: for each object, the proportion of 40 images labeled as normal/pathological exceeds 50%.

## Conclusion and future work

In this study, we propose MMIF that fuses image and text modalities, models multimodal data information, generates encoded unimodal image features, decoded unimodal text features, and multimodal decoding features, and finally diagnoses fetal well-being. The following key points were identified in our study:

1) Initially, our proposed MMIF combines two important network modules of CCPViT and MRAN to explore multimodal learning tasks and solve the misalignment problem. Specifically, sample labels were introduced first to construct unimodal text-only data. Then, we designed a constraint term loss for comparison learning with the image modality of CCPViT, and a captioning loss for auxiliary aligning with the multimodal fusion features of MRAN. Structurally, CCPViT takes ViT and Transformer as backbones and calculates the unimodal information of image and text modalities in parallel. Based on CCPViT, a cross-attention-based image–text joint component was further established to explore the deep-level causality and inclusion between cross-modal data and realize multimodal learning.
2) Furthermore, we designed a simple-structured FDD test model based on the highly modal alignment MMIF, realizing the task delegation from multimodal model training (image and text) to unimodal pathological diagnosis (image) and satisfying the constraints of data from different source domains in clinical tasks.
3) The proposed MMIF and its downstream model, i.e., the FDD test model, were verified on a public clinical database. Extensive experiments, including parameter sensitivity analysis, cross-modal alignment assessment, and pathological diagnostic accuracy evaluation, were conducted to show their superior performance and effectiveness.

Some interesting points in this study can be expanded. The first and most important problem is how to rigidly constrain the MMIF’s cross-modal alignment and evaluate its alignment effect in real time during multimodal learning. Another problem is insufficient model interpretability. A qualified medical diagnosis system must be transparent, understandable, and explainable. Thus, for future work, we will explore explainable AI models.

## Data Availability

The original contributions presented in the study are included in the article/Supplementary material, further inquiries can be directed to the corresponding author.
